# Otolaryngological Problems Among Patients with Growth Hormone Deficiency—A Systematic Review

**DOI:** 10.3390/jcm14093064

**Published:** 2025-04-29

**Authors:** Gazala Abdulaziz-Opiela, Paweł Witkowski, Yasmina Późniak, Julia Bajdor, Joanna Bautembach, Małgorzata Myśliwiec, Bogusław Mikaszewski

**Affiliations:** 1 Department of Otolaryngology, Faculty of Medicine, Medical University of Gdansk, 80-210 Gdansk, Poland; 2 Department of Opthalmology, Faculty of Medicine, Collegium Medicum, Nicolaus Copernicus University, 85-067 Bydgoszcz, Poland; 3 Department of Radiology, Nicolaus Copernicus Hospital, 80-803 Gdansk, Poland; 4 Department of Pediatrics, Diabetology and Endocrinology, Faculty of Medicine, Medical University of Gdansk, 80-210 Gdansk, Poland

**Keywords:** growth hormone, growth hormone deficiency, otorhinolaryngological problems

## Abstract

**Background/Objectives**: Growth hormone deficiency (GHD) is a rare endocrine disorder characterized by inadequate secretion of growth hormone, which affects growth, cellular processes, and physiological functions. In addition to growth impairment, GHD is associated with a range of otorhinolaryngological (ENT) symptoms, such as sensorineural hearing loss, dizziness, and voice alterations. These symptoms may be underrecognized due to a lack of routine ENT evaluations in GHD management. **Methods**: This systematic review, conducted in accordance with PRISMA guidelines, assessed the prevalence of ENT symptoms in patients with GHD by analyzing studies from the PubMed, Embase, and Scopus databases. **Results**: Based on the analysis of eleven studies that met the inclusion criteria, more than half of patients with GHD experience ENT symptoms (61.4%). Symptom variability appeared to correlate with treatment access, age of onset, and the presence of comorbidities. The study underscores the importance of routine ENT assessments as part of the multidisciplinary management of patients with GHD. **Conclusions**: Early detection and management of ENT symptoms may significantly improve quality of life, reduce social and educational challenges, and support long-term health outcomes. Further research is needed to clarify the role of recombinant human growth hormone therapy in mitigating ENT manifestations in this patient population.

## 1. Introduction

Growth hormone deficiency (GHD) is a rare endocrine disorder characterized by insufficient secretion of growth hormone (GH) from the anterior lobe of the pituitary gland. It is mainly responsible for growth, cell reproduction, and regeneration. Moreover, GH signaling triggers the production of insulin-like growth factor 1 (IGF-1) in hepatic and peripheral tissues, which facilitates diverse physiological processes [1].

Beyond the well-documented impact on growth, patients with GHD experience a wide spectrum of otorhinolaryngological (ENT) complications, such as a higher incidence of sensorineural hearing loss, misophonia, dizziness, laryngopharyngeal reflux, and high-pitched voice. However, the prevalence of these ENT manifestations remains challenging to determine, likely due to factors such as age of onset, severity of the deficiency, access to medical help, timing of treatment initiation, and the presence of comorbidities.

Despite the clinical significance of ENT complications in GHD, otorhinolaryngological evaluations are not routinely performed among these patients, resulting in underrecognition of many of these conditions. This systematic review aims to evaluate the prevalence of ENT conditions among patients with GHD to highlight the importance of routine ENT consultations as part of the multidisciplinary management of these patients.

## 2. Materials and Methods

### 2.1. Search Strategy

To examine the prevalence of otorhinolaryngological conditions among patients with growth hormone deficiency, a systematic review was conducted following the Preferred Reporting Items for Systematic Reviews and Meta-Analyses (PRISMA) guidelines [2]. A comprehensive search was performed across three major databases—PubMed (Medline), Scopus (Elsevier), and Embase (Elsevier)—to target relevant studies published between 1971 and 2024.

### 2.2. Searching and Data Screening

**Figure 1:**
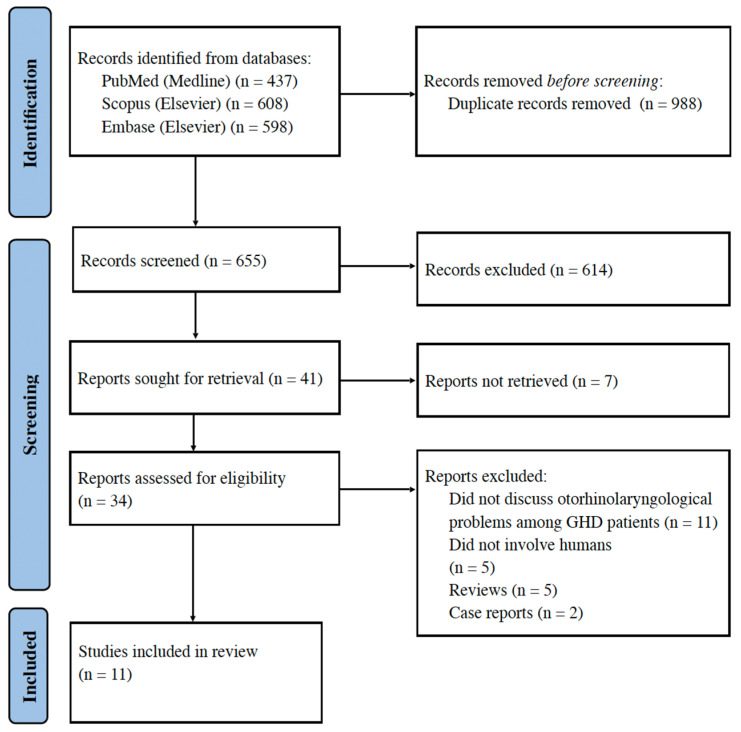
PRISMA flowchart.

On 8 October 2024, two independent authors conducted a manual database search. The search included specific terms, such as “hearing loss” OR “auditory” OR “voice” OR “otorhinolaryngology” OR “ENT” OR “tinnitus” OR “vertigo” AND “growth hormone deficiency” OR “GHD” OR “IGF-1 deficiency”. The initial search yielded 1643 articles, from which 988 duplicates were removed. Of the remaining 655 articles, 614 were excluded, primarily due to insufficient GHD data or non-human studies. Forty-one articles were assessed for eligibility; however, only eleven met the inclusion criteria. The process is detailed in the PRISMA flowchart in Figure 1. Any disagreements were resolved by consensus.

### 2.3. Eligibility Criteria

This systematic review used a structured PICOTS framework (Population, Intervention, Comparison, Outcome, Time, and Setting) [3]. The population included patients diagnosed with GHD, while the interventions were ENT consultations and relevant diagnostic tests. The primary outcome was the incidence of ENT conditions in this population. The timeframe considered was the period following GHD diagnosis. The setting encompassed evaluations by otolaryngologists and endocrinologists involved in the management of patients with GHD. Specific inclusion and exclusion criteria are detailed in Table 1.

### 2.4. Data Extraction and Processing

Data were manually extracted from each included article, focusing on several essential parameters: (a) study details, including authors and publication year; (b) the number of patients diagnosed with growth hormone deficiency; (c) the incidence of otorhinolaryngological problems within the cohort; and (d) type of ENT symptoms.

To evaluate the quality of each study, we used the Newcastle–Ottawa Scale [4], which is designed for non-randomized observational studies, including cohort and case–control designs. This scale assigns up to nine stars in three areas: selection (four stars), comparability (two stars), and outcomes (three stars). Studies scoring at least seven stars are considered to have a low risk of bias, reflecting high methodological quality.

## 3. Results

### 3.1. Databases Search Results

A manual search of the mentioned databases yielded 655 results. Following the title and abstract screening, 614 articles were excluded as they did not meet the PICOTS criteria. Subsequently, 41 articles were qualified for full-text assessment, however, 7 articles could not be retrieved. Of the remaining 34 articles, 23 were excluded for various reasons: 11 did not discuss otorhinolaryngological problems in patients with GHD, 5 did not involve humans, 5 were reviews, and 2 were case reports. Ultimately, 11 articles met the criteria for this systematic review.

### 3.2. Included Studies Characteristics

Moore et al. [5] conducted a study on adult males with congenital isolated growth hormone deficiency (IGHD) from Pakistan, comparing their findings with four patients who had acquired GHD. The research demonstrated that individuals with congenital GHD exhibited high-pitched and raspy voice characteristics typical of normal females (174–266 Hz), while three out of four patients with adequate GHD levels during puberty had voices within the normal frequency range.

Valença et al. [6] aimed to evaluate the quality of voice in 33 patients with IGHD from Itabaianinha County, Brazil. The results indicated that individuals with IGHD had significantly lower Voice-Related Quality-of-Life (V-RQOL) scores and higher fundamental frequency compared to the control group, suggesting alterations in vocal perception and characteristics.

In a subsequent study, Valença et al. [7] aimed to assess voice formant frequencies (F1, F2, F3, F4) by performing acoustic analysis. The analysis revealed that IGHD males exhibited elevated F3 and F4 values for specific vowels, while IGHD females showed increased F1 and F4 values and decreased F2 values for certain vowels compared to controls. These findings suggest that congenital IGHD leads to elevated formant frequencies and results in a prepubertal acoustic profile. These patients were excluded from the overall prevalence count as they belonged to the same study group as the previous study.

Prado-Barreto et al. [8] assessed auditory function in 26 adults from Itabaianinha County, Brazil. Compared to controls, IGHD patients reported higher incidences of misophonia and dizziness and exhibited mild high-frequency sensorineural hearing loss, absent stapedial reflexes, and absent transient evoked otoacoustic emissions (TEOAEs). Hearing impairment occurred earlier in IGHD patients than in controls.

Barreto et al. [9] evaluated the effects of untreated IGHD on vocal and laryngeal function in 23 adults with IGHD compared to 22 controls. IGHD subjects exhibited a higher incidence of vocal abnormalities, including roughness, breathiness, and strain, as well as increased signs of laryngopharyngeal reflux and laryngeal constriction, despite similar vocal complaints between groups.

Seventeen of these patients were included in two studies conducted by de Andreade et al. The first examined the effects of semi-occluded vocal tract therapy (SOVTT) and choir training on voice. Acoustic analysis of isolated vowels demonstrated an increase in the first formant following choir training, while the second formant increased after SOVTT. Choir training showed a tendency to reduce shimmer, reflecting improved vocal stability [10]. The second study by de Andreade et al. evaluated the impact of voice therapy on voice quality [11]. After a 13-week intervention, patients demonstrated significant improvements in V-RQOL and self-assessment of voice quality. Reductions in vocal deviations, including roughness, breathiness, and strain, were also observed. These patients were excluded from the overall prevalence count as they belonged to the same study group as the previous study.

Santos-Carvalho et al. [12] assessed vestibular function in 15 adults with untreated IGHD, who exhibited a higher prevalence of abnormalities in clinical head impulse and oculomotor tests, particularly in saccadic eye movements and spontaneous nystagmus, when compared to controls, indicating possible vestibular impairment.

Kocyigit et al. [13] conducted a study involving 207 patients with various endocrine disorders, including 46 with GHD, to evaluate auditory symptoms such as hearing loss, tinnitus, and vertigo. Each participant underwent tympanometry and pure-tone average (PTA) and high-frequency average (HFA) audiometry. Patients with conductive hearing loss were excluded from the study. Among the GHD group, five patients were diagnosed with sensorineural hearing loss (out of 46 patients, 1 had a PTA value > 20 dB, 3 had HFA value > 20 dB, and 1 had both PTA and HFA values > 20 dB). Seven patients reported tinnitus, and three reported experiencing vertigo. Notably, vertigo was assessed solely through the Pediatric Vestibular Symptom Questionnaire, and videonystagmography was not performed. Tinnitus was also evaluated exclusively via questionnaire. These methodological aspects may represent limitations in the comprehensive assessment of vestibular and auditory symptoms. No significant differences in symptom prevalence were observed when compared to the control group.

In a separate study, Kocyigit et al. [14] assessed the prevalence of otitis media with effusion (OME) in 918 pediatric patients with endocrine disorders, including 179 with GHD. OME was found to be significantly more prevalent in patients with GHD (30.2%) compared to the control group.

Muus et al. [15] investigated the prevalence, type, and severity of hearing impairment in a cohort of children with growth hormone deficiency. Auditory assessment included pure-tone audiometry and tympanometry. The study enrolled 209 children with GHD, of whom 173 (83%) demonstrated hearing loss. Bilateral involvement was observed in 79% of cases, while 21% were unilateral. Due to incomplete audiometric data, a substantial proportion of patients were categorized as having undefined hearing loss (53.6%). Among those with a defined diagnosis, 65 children were found to have mixed hearing loss. Pure conductive hearing loss was identified in 47 patients, and pure sensorineural hearing loss in 24 patients.

### 3.3. Risk of Bias Assessment

Each article was carefully assessed using the Newcastle–Ottawa Scale [4] to evaluate methodological quality and identify potential biases. All included studies demonstrated a low risk of bias. However, a source of bias was identified, as many studies concentrated on individuals with untreated congenital IGHD linked to the homozygous mutation in the GH-releasing hormone receptor (GHRHR) gene from Itabaianinha County, Brazil. In this population, otorhinolaryngological symptoms observed were more severe than in treated patients. Additionally, since multiple articles originated from the same research group, they were counted only once in the final prevalence analysis to minimize bias.

Moreover, some patients presented with multiple otorhinolaryngological symptoms, complicating the accurate estimation of the total number affected. In such cases, we recorded only the most prevalent symptom, which may have resulted in an underestimation of the overall prevalence of individuals impacted.

### 3.4. Results of Data Synthesis

A total of 539 patients diagnosed with GHD who underwent otorhinolaryngological examination were included in this systematic review. The majority of patients in the study experienced various ENT problems (331 patients, 61.4%).

Following the exclusion of patients with congenital IGHD associated with a homozygous mutation in the GHRHR gene, 234 of the remaining 434 patients (53.9%) were found to exhibit ENT-related symptoms.

Comprehensive data regarding these patients, along with all important information extracted from the included articles, are presented in Table 2. Results of the data synthesis are presented in Table 3.

## 4. Discussion

Growth hormone is a 191 amino acid peptide produced by somatotroph cells of the anterior lobe of the pituitary gland. GH receptors are widely distributed, particularly in the liver, as well as in cartilage, muscle, adipose tissue, and the kidneys. Upon binding to its receptors, GH initiates a cascade of intracellular signals that activate Janus kinase 2 (JAK2) and signal transducer and activator of transcription (STAT) proteins. This activation regulates specific target genes, primarily stimulating the liver to produce IGF-1. Both GH and IGF-1 are essential for chondrocyte proliferation and linear growth. However, other hormones, such as sex steroids and thyroid hormones, also play significant roles in these processes. Additionally, GH facilitates lipolysis, leading to the release of free fatty acids, a reduction in total cholesterol and apolipoprotein B levels, and an increase in high-density lipoprotein levels. Furthermore, GH stimulates osteoblast differentiation, proliferation, and bone formation, while IGF-1 is particularly necessary to maintain cortical bone repair and remodeling [1].

Growth hormone deficiency can be classified by age of onset (pediatric or adult), etiology (congenital, acquired, or idiopathic), and severity (isolated deficiency or as part of multiple pituitary hormone deficiencies). Pediatric cases are often isolated and idiopathic. In adults, acquired growth hormone deficiency (AGHD) is most commonly due to pituitary or hypothalamic tumors, with pituitary adenomas and craniopharyngiomas accounting for more than half of AGHD cases. AGHD may also result from treatments such as surgery or radiation therapy [16,17].

The diagnosis of GHD involves an assessment of growth patterns, clinical examination, and laboratory evaluations, including GH provocative testing (using pharmacologic agents such as insulin, clonidine, arginine, or glucagon) and IGF-1 measurements. Imaging studies, particularly MRI of the hypothalamic–pituitary region, are performed to detect any structural abnormalities, such as pituitary tumors, pituitary hypoplasia, or other lesions that may explain GHD. A detailed medical history, including prenatal and family history, is also collected to identify possible genetic or acquired risk factors for GHD. Early diagnosis and treatment with recombinant human growth hormone are essential to address growth delays in children, ensuring they achieve their optimal adult height and alleviating other systemic effects [18].

Individuals with untreated GHD frequently experience symptoms affecting multiple organs. Research on individuals from Itabaianinha County, Brazil, who have severe congenital IGHD due to a homozygous GHRHR gene mutation and have never received treatment shows significant physical consequences such as severe short stature (ranging from 105 to 135 cm), central obesity, dyslipidemia, and elevated systolic blood pressure. Despite these substantial health challenges, they report a strong sense of well-being and satisfaction with their quality of life, possibly due to the support of their community [8,19]. Due to the fundamental roles of GH and IGF-1 in the development of all body organs, the deficiency may lead to various otorhinolaryngological symptoms. Growth hormone and insulin-like growth factor 1 are essential regulators of inner ear development and auditory function. Research on murine models has shown that GH plays a critical role in the embryonic development of the inner ear, shown by significant expression of this hormone in the cochlear sensory epithelium during postnatal growth [20].

According to a study conducted by Gomez et al., prolonged exposure to high-intensity noise can lead to an initial increase in circulating GH levels, suggesting a potential neuroprotective role and facilitation of neuroregeneration [21]. GH replacement therapy in individuals with brain injuries has also shown promise in restoring hearing by promoting the regeneration of cochlear sensory cells and the auditory nerve [21].

IGF-1 is also crucial in the survival and proliferation of inner ear cells. It reaches the inner ear through systemic circulation and is locally expressed in the cochlear and vestibular ganglia, providing protection against apoptosis. Moreover, circulating levels of IGF-1 are directly correlated with the severity of hearing loss, and topical administration of IGF-1 has been associated with positive outcomes in the recovery from sudden sensorineural hearing loss [22].

In comparison to healthy individuals, patients with untreated IGHD demonstrate significantly higher hearing thresholds across all frequencies and are more likely to experience mild high-frequency hearing loss. Additionally, the absence of stapedial reflex and TEOAEs has been observed, along with complaints of dizziness and misophonia, both of which occur more commonly in IGHD patients. Vestibular impairments are also notable, as head impulse testing often reveals saccadic eye movements and spontaneous nystagmus, suggesting compromised vestibular function in IGHD patients [8,12].

Research has also evaluated patients receiving recombinant human growth hormone therapy. In a study conducted on pediatric patients with endocrine disorders, Kocyigit et al. observed sensorineural hearing loss, tinnitus, and vertigo among GHD patients, though the findings were not significant compared to healthy controls. Nonetheless, the study indicated a subtle negative impact on inner ear functions, suggesting that even treated GHD patients may experience some otological challenges [13].

Otitis media with effusion (OME) is a primary cause of hearing impairment in children, though its prevalence among pediatric patients with endocrine conditions has not been well documented. In a study involving 918 patients, including 179 with GHD, Kocyigit et al. reported that children with endocrine conditions are more likely to suffer from OME than their healthy peers [14]. However, the underlying mechanisms remain unclear, highlighting the need for further research.

Auditory challenges may impact vocal development, which is also influenced by other factors, such as the geometry of the vocal tract, nasal and maxillary structures, and mandibular length. As individuals mature, growth of the larynx and vocal folds generally leads to a reduction in fundamental frequency (f0). Pediatric patients typically have a high f0 regardless of gender, but as they mature, males experience a lowering of pitch, while females maintain a higher pitch throughout adulthood until post-menopause [6].

However, untreated patients with IGHD have a higher fundamental frequency (f0) and do not undergo the typical age-related changes in voice observed in the general population. These patients often present with qualities such as shimmer and breathiness which can be caused by the small laryngeal size and impaired vocal fold development due to the absence of growth hormone during puberty [6]. Additionally, underdeveloped nasopharyngeal cavities may distort vocal tonality. These findings emphasize the importance of early diagnosis and treatment to address voice abnormalities and enhance quality of life in patients with GHD [5].

On the other hand, individuals with acquired GHD, which is often caused by pituitary tumors, usually maintain a voice frequency within the normal male range, indicating preserved laryngeal structure. This suggests that the timing of GHD onset significantly impacts vocal characteristics [5].

Patients with untreated IGHD exhibit a higher prevalence of laryngopharyngeal reflux, laryngeal constriction, and vocal abuse, likely because of the proximity of the esophagus and related structures associated with their small stature [9]. Due to their altered vocal characteristics, these patients have lower V-RQOL scores compared to healthy controls. However, interventions such as semi-occluded vocal tract therapy and choir training have demonstrated improvements in voice parameters, including reductions in roughness, breathiness, and strain, contributing to an overall increase in V-RQOL [10,11].

Several limitations should be considered when interpreting the findings of the included studies. Hearing loss was assessed primarily through audiometry and tympanometry, while tinnitus was evaluated exclusively via questionnaire-based methods, which may have reduced the accuracy of symptom assessment. Moreover, none of the studies evaluated the progression of hearing impairment over time. Some studies also included patients with untreated congenital isolated GHD that may not reflect the current standard of care. Given the increasing availability and early initiation of recombinant human growth hormone therapy, which is likely to alleviate some of the associated ENT manifestations, the prevalence of symptoms reported in these studies may be overestimated.

However, knowledge of otorhinolaryngological manifestations in growth hormone deficiency remains limited, underscoring the need for more comprehensive research in this area. Patients with GHD may experience a reduced quality of life due to various ENT-related issues, which can negatively impact academic performance and limit access to appropriate support in educational settings. Certain otorhinolaryngological interventions can be implemented to address these issues. For instance, tympanostomy placement can help alleviate middle ear problems by facilitating drainage and ventilation. Additionally, the implementation of hearing aids for those with sensorineural hearing loss can significantly enhance auditory function and communication abilities. Hearing impairments may also contribute to speech and language delays, necessitating the involvement of speech-language therapists. By addressing auditory deficits and improving communication skills, these interventions can help children with GHD perform better academically and socially.

## 5. Conclusions

In summary, otorhinolaryngological symptoms affect more than half of individuals with growth hormone deficiency, underscoring the necessity for routine ENT consultations. Early identification and intervention can enhance quality of life and mitigate the complications associated with GHD. Collaboration among healthcare professionals is essential to provide optimal care for these patients. Furthermore, additional research is needed to assess the prevalence of otorhinolaryngological conditions among patients diagnosed in childhood who are qualified for treatment, as well as the impact of recombinant human growth hormone therapy on otorhinolaryngological symptoms.

## Figures and Tables

**Table 1:** Inclusion and exclusion criteria for the selection of articles.

Inclusion Criteria	Exclusion Criteria
Patients with the diagnosis of GHD who underwent ENT examination at any time	Patients with short stature due to genetic conditions, e.g., Turner syndrome, Prader–Willi syndrome
	Case report/case series

**Table 2:** Relevant data extracted from the articles.

First Author Name	Number of Patients with Growth Hormone Deficiency	Number of Patients with Otorhinolaryngological Symptoms	Symptoms Experienced by Patients	Newcastle–Ottawa Scale (*n*)
Moore et al. [5]	8	5	High-pitched voice	7
Valença et al. [6]	33	33	High-pitched voice	7
Valença et al. [7] *	33	33	High-pitched voice	7
Prado-Barreto et al. [8]	26	26	Hearing loss, dizziness, misophonia	7
Baretto et al. [9]	23	23	Laryngopharyngeal reflux, vocal abnormalities	7
de Andreade et al. [10] *	17	17	High-pitched voice	7
de Andreade et al. [11] *	17	17	High-pitched voice	7
Santos-Carvalho et al. [12]	15	10	Vestibular impairment	8
Kocyigit et al. [13]	46	7	Hearing loss, tinnitus, vertigo	8
Kocyigit et al. [14]	179	54	Otitis media with effusion	8
Muus et al. [15]	209	173	Hearing loss	8

* not included in overall prevalence count.

**Table 3:** The prevalence of ENT conditions among patients with GHD.

Otorhinolaryngological Symptoms	N (%)
Yes	331 (61.4)
No	208 (38.6)

## Data Availability

The raw data supporting the conclusions of this article will be made available by the authors without undue reservation.

## Abbreviations

The following abbreviations are used in this manuscript:

AGHD	Acquired growth hormone deficiency
GH	Growth hormone
GHD	Growth hormone deficiency
GHRHR	GH-releasing hormone receptor
HFA	High frequency averages
IGF-1	Insulin-like growth factor 1
JAK2	Janus kinase 2 protein
OME	Otitis media with effusion
ENT	Otorhinolaryngological
PTA	Pure-tone averages
SOVTT	Semi-occluded vocal tract therapy
TEOAEs	Transient evoked otoacoustic emissions
V-RQOL	Voice-Related Quality-of-Life
